# Prevalence of Plasmid-Mediated Determinants With Decreased Susceptibility to Azithromycin Among *Shigella* Isolates in Anhui, China

**DOI:** 10.3389/fmicb.2020.01181

**Published:** 2020-06-30

**Authors:** Yanyan Liu, Hongru Li, Na Lv, Yalong Zhang, Xihai Xu, Ying Ye, Yufeng Gao, Jiabin Li

**Affiliations:** ^1^Department of Infectious Diseases, The First Affiliated Hospital of Anhui Medical University, Hefei, China; ^2^Anhui Center for Surveillance of Bacterial Resistance, Hefei, China; ^3^Institute of Bacterial Resistance, Anhui Medical University, Hefei, China; ^4^Department of Neurology, Xiangya Hospital Central South University, Changsha, China; ^5^Department of Infectious Diseases, The Chaohu Affiliated Hospital of Anhui Medical University, Hefei, China

**Keywords:** *Shigella*, plasmid, azithromycin, multidrug-resistant, PFGE

## Abstract

**Objective:**

The aims of this study were to describe azithromycin (AZM) susceptibility patterns among *Shigella* isolates in Anhui, China and identify predictors of resistance with mobile element-mediated genes.

**Methods:**

A total of 517 non-duplicate *Shigella* isolates (449 *S. flexneri* and 68 *S. sonnei*) were collected in the Anhui Province of China from September 2011–September 2015, and screened for the plasmid-mediated genes of decreased susceptibility to AZM (DSA), using polymerase chain reaction amplification and sequencing. Conjugation experiments and pulsed-field gel electrophoresis were conducted for all *mphA-*positive DSA isolates.

**Results:**

The DSA rate for 449 *S. flexneri* isolates was 33.6%, compared with 39.7% for 68 *S. sonnei* isolates. Among 161 DSA *S. flexneri* isolates, 93 (57.8%) carried the *mphA* gene. Among 27 DSA *S. sonnei* isolates, 11 (40.7%) carried the *mphA* gene. However, other plasmid-mediated DSA genes were not found in these isolates. A total of 89 transconjugants (95.7%) were obtained from 93 *mphA*-positive *S. flexneri* isolates through conjugation, and 10 transconjugants (90.9%) were obtained from 11 *mphA*-positive *S. sonnei* isolates. Furthermore, the minimum inhibitory concentrations (MICs) of AZM among 89 *S. flexneri* transconjugants ranged from 4 to 128 μg/mL, with an MIC_50_ of 8 μg/mL and MIC_90_ of 32 μg/mL. The MICs of AZM among 10 *S. sonnei* transconjugants ranged from 4 to 256 μg/mL, with an MIC_50_ of 8 μg/mL and MIC_90_ of 64 μg/mL. Thirteen clusters were found for *mphA*-positive *S. flexneri*, and five clusters were found for *mphA*-positive *S. sonnei*. Furthermore, 10 homologous isolates among 13 *mphA*-positive *S. flexneri* isolates with high-level DSA were from Sixian county and were multidrug-resistant strains. Of the 10 homologous *S. flexneri* isolates, eight were from children (≤5 years old), and two from the elderly (>60 years old).

**Conclusion:**

Our study demonstrates that the DSA for *Shigella* isolates was severe, and the plasmid-mediated *mphA* gene was the most common macrolide resistance gene detected in *Shigella* isolates collected in Anhui, China. The *mphA*-positive *S. flexneri* isolates with high-level DSA facilitated clonal spread in children and the elderly. This finding is noteworthy and warrants further study.

## Introduction

Shigellosis is an acute invasive enteric infection caused by bacteria of the genus *Shigella*, including *S. dysenteriae*, *S. flexneri*, *S. boydii*, and *S. sonnei*. The symptoms of shigellosis usually include fever, headache, vomiting, and abdominal cramps, followed by watery diarrhea. All species of *Shigella* could be spread by direct contact with an infected person or via contaminated objects, food, or water, as well as close personal or sexual contact with fecal exposure (Kotloff et al., 2018).

Although shigellosis is typically self-limiting, treatment with appropriate antimicrobial therapy can shorten the duration of illness and prevent transmission. In guidelines published by the WHO in 2005, ciprofloxacin (CIP) was considered the first-line treatment for shigellosis, with ceftriaxone (CRO) and azithromycin (AZM) listed as alternative options. However, widespread use of CIP and third-generation cephalosporins have led to increasing resistance to these antimicrobials among *Shigella* isolates, especially in Asia (Liu et al., 2012, 2017; Zhang et al., 2014; Kotloff et al., 2018). The American Academy of Pediatrics and the Infectious Diseases Society of America have recommended the use of AZM to treat multidrug-resistant shigellosis (Erdman et al., 2008; Pickering et al., 2009; Shane et al., 2017). In China, AZM is infrequently used to treat shigellosis. Thus, there is a relative lack of published research on decreased susceptibility to AZM (DSA) for *Shigella* in China.

As a macrolide antibiotic, AZM has been used primarily to treat Gram-positive bacterial infections, and has shown favorable effects against various Gram-negative organisms (Gomes et al., 2013b). However, there are currently no interpretive criteria for AZM susceptibility testing of *Shigella*. According to the Clinical and Laboratory Standards Institute guidelines (CLSIs) published in 2019, epidemiological cut-off values (ECVs) of minimum inhibitory concentrations (MICs) ≥ 16 μg/mL (for *S. flexneri*) and MIC ≥ 32 μg/mL (for *S. sonnei*) were considered indicative of DSA. Previous studies on DSA have been focused mainly on punctual mutations in the *rplD* (encoding the L4 ribosomal protein), *rplV* (encoding the L22 ribosomal protein), and *rrlH* (23S rRNA) genes, and chromosomal efflux pumps (such as OmpA and OmpW) (Gomes et al., 2013a; Campbell et al., 2019).

More recently, studies have demonstrated that DSA-*Shigella* isolates in many areas are closely associated with the plasmid-borne *mphA* gene, which encodes macrolide-2′-phosphotransferase mediating macrolide resistance (Gaudreau et al., 2014; Darton et al., 2018; Yousfi et al., 2019). Besides the *mphA* gene, other mobile genetic elements also play an important role in the development of DSA, including *mphB*, *ermA*, *ermB*, *ermC*, *ereA*, *ereB*, *mefA*, *msrA*, *ermF*, *ermT*, and *ermX*) (Belkacem et al., 2016; Salah et al., 2019). Owing to the capacity for horizontal transmission among bacteria, the prevalence of mobile element-located resistance genes is a considerable challenge in controlling the spread of DSA.

To the best of our knowledge, the susceptibility of *Shigella* to AZM has not yet been recorded in Anhui, China. Therefore, the aims of this study were to describe AZM susceptibility patterns among *Shigella* isolates in Anhui, China and identify predictors of resistance with the mobile element-mediated genes.

## Materials and Methods

### Bacteria Isolates

A total of 517 non-duplicate *Shigella* isolates (449 *S. flexneri* and 68 *S. sonnei*) were collected from the fecal samples of various patients at 34 hospitals in the Anhui Province of China, between September 2011 and September 2015. Among 517 patients with shigellosis, 506 patients lived in Anhui, eight patients lived in another province, and the original location of three patients was unknown. None of these patients had taken AZM within the previous 3 months. Individual isolates were identified by using standard microbiological and biochemical methods. All *Shigella* isolates were confirmed by using an API-20E system (bioMérieux, Marcy l’ Étoile, France) and serotyped by using commercial antisera (Denka Seiken Co., Ltd., Tokyo, Japan). Strains of *Escherichia coli* ATCC 25922, *Salmonella* H9812, and sodium azide-resistant *Escherichia coli* J53 (*E. coli* J53Az^*R*^) were stored at the Anhui Center for Surveillance of Bacterial Resistance (Hefei, Anhui, China).

The study was conducted in accordance with the guidelines of the Declaration of Helsinki, the principles of Good Clinical Practice, and Chinese regulatory requirements, and was approved by the local Ethics Committees of the First Affiliated Hospital of Anhui Medical University (Hefei, China). All patients gave written informed consent.

### Antimicrobial Agents

All antibiotics were obtained from Sigma-Aldrich China (Shanghai, China). Bacteria were cultured to the log-phase (approximately 1.5 × 10^8^ CFU/mL). The MICs of AZM, CIP, CRO, and ampicillin (AMP) were determined by the agar dilution method using Mueller-Hinton agar (Oxoid Ltd., Basingstoke, United Kingdom) containing a series of twofold diluted antibiotics. The antibiotic plates with bacterial colonies were incubated at 37°C for 18–20 h. The results were interpreted according to CLSI breakpoints published in 2019. The susceptibility data of *Shigella* isolates were accepted only if the MIC for quality control strains, tested in parallel, was within the acceptable ranges given in the CLSI guidelines. The quality control strain was *E. coli* ATCC 25922.

### Detection of Plasmid-Mediated Resistance Genes

Bacterial DNA was extracted by the boiling method and plasmid DNA was extracted using the Qiagen Plasmid Purification kit (QIAGEN, Hilden, Germany). The plasmid-mediated DSA genes (*mphA/B*, *ermA/B/C/F/T/X*, *ereA/B*, *mefA*, and *msrA*) for DSA-*Shigella* isolates were identified through the polymerase chain reaction (PCR). Primer sequences are shown in Supplementary Table S1. Cycle conditions for polymerase activation were as follows: initial denaturation at 95°C for 3 min; then 30 cycles of amplification, including denaturation at 95°C for 30 s; annealing for 30 s (annealing temperature is presented in Supplementary Table S1); extension at 72°C for 1 min; and a single final extension at 72°C for 10 min. All PCR products were purified using the QIAquick PCR Purification Kit (QIAGEN, Hilden, Germany) and directly sequenced. Sequence alignments were compared with the GenBank nucleotide database to determine the plasmid-mediated AZM resistance genotype.

### Conjugation Experiments and Transfer of Drug Resistance

Conjugation experiments were conducted for all isolates positive for *mphA*, with E. coli J53AzR as the recipient. Transconjugants were selected on MacConkey agar plates supplemented with sodium azide (200 μg/mL) (Sigma Chemical Co., St. Louis, MO, United States), and AMP (32 μg/mL). Transconjugants were tested by the biochemical method and confirmed as *E. coli* using the API-20E system (bioMérieux). Plasmid DNA extraction from donors and transconjugants was performed using the Qiagen Plasmid Purification kit (QIAGEN, Hilden, Germany). The transconjugants were examined through PCR for the presence of the *mphA* gene using plasmid DNA as the template. The MICs of AZM, AMP, CIP, and CRO were determined for the recipient, donors, and transconjugants. The assays were conducted according to the methods described above.

### Pulsed-Field Gel Electrophoresis

The DNA fingerprinting profiles were analyzed by pulsed-field gel electrophoresis (PFGE) after digestion with the restriction enzyme, *Xba*I (Takara, Inc., Dalian, China), according to the procedures developed by the US Centers for Disease Control and Prevention (CDC) Pulse Net program. Electrophoresis was performed on 1% agarose gels in 0.5 M Tris/borate/EDTA buffer on a CHEF Mapper^®^ XA PFGE system (Bio-Rad, Hercules, CA, United States) for 22 h at 14°C, with run conditions of 6 V/cm, a pulse angle of 120°, and pulse times from 2.16 to 63.8 s. *Salmonella* H9812 was used as a molecular mass marker. The band profiles of PFGE were analyzed using the BioNumerics software (version 7.6, Applied Maths, sint-martens-latem, Belgium). Dendrograms of the band profiles were produced using the unweighted pair group method with mathematical averaging. The relatedness of isolates was calculated using the Dice coefficient with a band position tolerance setting of 1–1.5%. Isolates were defined as the same PFGE type (clonal) if the Dice coefficient was ≥ 85%.

### Statistical Analysis

Data were analyzed using the SPSS version 16.0 software (SPSS Inc., Chicago, IL, United States). Univariate analysis was performed using the chi-squared test or Fisher’s exact test, as appropriate. *P*-values were based on two-tailed test results, and *P* < 0.05 were considered statistically significant.

## Results

### Antimicrobial Susceptibility

The characteristics of patients with shigellosis and the antimicrobial resistance of 517 *Shigella* isolates are presented in Table 1. The DSA rate for 449 *S. flexneri* isolates was 33.6%, compared with 39.7% for 68 *S. sonnei* isolates (χ^2^ = 0.966, *P* = 0.33). No significant differences were noted in gender or age between *S. flexneri* and *S. sonnei* isolates with DSA. The CIP resistance rate for 449 *S. flexneri* isolates was 49.7%, compared with 19.1% for 68 *S. sonnei* isolates (χ^2^ = 22.213, *P* < 0.0001). Regarding CIP resistance, no significant differences were noted between *S. flexneri* and *S. sonnei* among male participants and all age groups, and similarly, no significant differences were noted among female participants. The CRO resistance rate for 449 *S. flexneri* isolates was 45.7%, compared with 33.8% for 68 *S. sonnei* isolates (χ^2^ = 3.355, P = 0.07). No significant differences were noted between *S. flexneri* and *S. sonnei*, regarding gender, age, and CRO resistance. However, significant difference regarding CRO resistance was noted in children under 5 years of age.

**TABLE 1:** Characteristics of patients with shigellosis and antimicrobial resistance of 517 *Shigella* isolates.

Risk factor	Total		DSA				Ciprofloxacin resistant				Ceftriaxone resistant			
				
	*S. flexneri*	*S. sonnei*	*S. flexneri*	*S. sonnei*	χ^2^	*p*-value	*S. flexneri*	*S. sonnei*	χ^2^	*p*-value	*S. flexneri*	*S. sonnei*	χ^2^	*p*-value
	(%)	(%)	(%)	(%)			(%)	(%)			(%)	(%)		
Total (*n* = 517)	449 (86.8)	68 (13.2)	151 (33.6)	27 (39.7)	0.966	0.33	223 (49.7)	13 (19.1)	22.213	<0.0001*	205 (45.7)	23 (33.8)	3.355	0.07
**Sex**														
Male	256 (57.0)	32 (47.1)	88 (34.4)	13 (40.6)	0.488	0.49	198 (77.3)	7 (21.9)	52.663	<0.0001*	120 (46.9)	10 (31.3)	2.804	0.09
Female	193 (43.0)	36 (52.9)	63 (32.6)	14 (51.9)	0.53	0.47	25 (13.0)	6 (16.7)		0.60^#^	85 (44.0)	13 (36.1)	0.779	0.38
**Age**														
Under 5 years	289 (64.4)	43 (63.2)	99 (34.3)	15 (34.9)	0.007	0.94	234 (81.0)	7 (16.3)	78.726	<0.0001*	141 (48.8)	14 (32.6)	3.962	0.047*
5–19 years	54 (12.0)	15 (22.1)	20 (37.0)	8 (53.3)	1.293	0.26	43 (79.6)	4 (26.7)		**<0.0001^#^**	26 (48.1)	6 (40.0)	0.313	0.58
20–59 years	44 (9.8)	7 (10.3)	9 (20.5)	2 (28.6)		0.64^#^	34 (77.3)	1 (14.3)		**0.003^#^**	16 (36.4)	1 (14.3)		0.41^#^
60 years and over	53 (11.8)	3 (4.4)	18 (34.0)	1 (33.3)		1^#^	46 (86.8)	0		**0.004^#^**	23 (43.4)	1 (33.3)		1^#^

DSA, decreased susceptibility to azithromycin. ^#^Fisher’s exact test. *Chi-square test.

**FIGURE 1:**
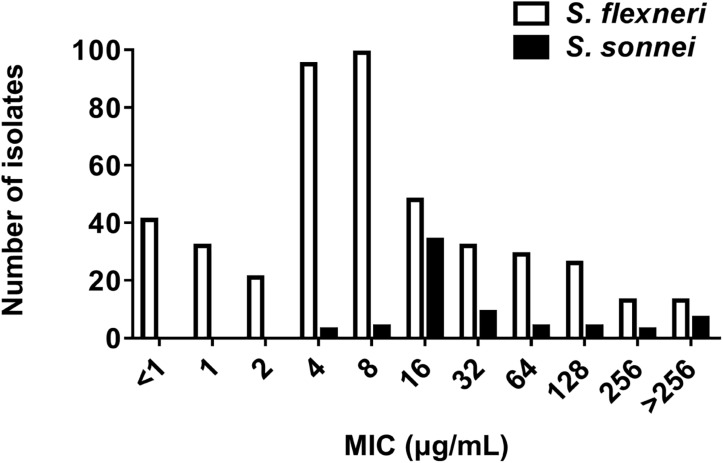
Azithromycin MIC by species. MIC frequency of azithromycin for *S. flexneri* (449 isolates, MIC_50_ = 4 μg/mL; MIC_90_ = 128 μg/mL) and *S. sonnei* (68 isolates, MIC_50_ = 16 μg/mL; MIC_90_ > 256 μg/mL).

The MIC frequencies of AZM for *S. flexneri* and *S. sonnei* are presented in Figure 1. The MICs of AZM among 449 *S. flexneri* isolates ranged from < 1 to > 256 μg/mL, with an MIC_50_ of 4 μg/mL and MIC_90_ of 128 μg/mL. The MICs among 68 *S. sonnei* isolates ranged from 4 to > 256 μg/mL, with an MIC_50_ of 16 μg/mL and MIC_90_ of > 256 μg/mL.

### Prevalence of Plasmid-Mediated DSA Genes

Among 161 DSA *S. flexneri* isolates, 93 (57.8%) carried the *mphA* gene. Among 27 DSA *S. sonnei* isolates, 11 (40.7%) carried the *mphA* gene. However, other plasmid-mediated DSA genes (such as *mphB, ermA, ermB, ermC, ermF, ermT, ermX, ereA, ereB, mefA*, and *msrA*) were not detected among these isolates. The MIC frequency of AZM for *mphA*-positive Shigella isolates are presented in Table 2. Of the 93 *mphA*-positive *S. flexneri* isolates, 13 (14%) exhibited high-level DSA (MIC > 256 μg/mL). Of the 11 *mphA*-positive *S. sonnei* isolates, two (18.2%) exhibited high-level DSA.

**TABLE 2:** MIC frequency of azithromycin for *mphA*-positive DSA *Shigella*.

Isolate (n)	MIC					
	
	>256 μg/mL	256 μg/mL	128 μg/mL	64 μg/mL	32 μg/mL	16 μg/mL
	(%)	(%)	(%)	(%)	(%)	(%)
*mphA*-DSA *S. flexneri* (93)	13 (14.0)	6 (6.5)	20 (21.5)	17 (18.2)	15 (16.1)	22 (23.7)
*mphA*-DSA *S. sonnei* (11)	2 (18.2)	0	2 (18.2)	2 (18.2)	5 (45.4)	–

*DSA, decreased susceptibility to azithromycin.*

### Transferability of Resistance Genes and Antimicrobial Susceptibility for Transconjugants

A total of 89 transconjugants (95.7%) were obtained from 93 *mphA*-positive *S. flexneri* isolates by conjugation, while 10 transconjugants (90.9%) were obtained from 11 *mphA*-positive *S. sonnei* isolates. The MICs of AZM among 89 transconjugants from *S. flexneri* isolates ranged from 4 to 128 μg/mL, with an MIC_50_ of 8 μg/mL and MIC_90_ of 32 μg/mL (Table 3). The MICs of AZM among 10 transconjugants from *S. sonnei* isolates ranged from 4 to 256 μg/mL, with an MIC_50_ of 8 μg/mL and MIC_90_ of 64 μg/mL (Table 4).

**TABLE 3:** MIC of 4 antimicrobial agents for *S. flexneri*, recipient strains and transconjugants.

Antimicrobial agent	*S. flexneri* (*n* = 89)			*E. Coli* J53 AzR	Transconjugants (*n* = 89)		
			
	MIC range	MIC_50_	MIC_90_		MIC range	MIC_50_	MIC_90_
	(μg/mL)	(μg/mL)	(μg/mL)		(μg/mL)	(μg/mL)	(μg/mL)
AZM	16 – > 256	64	>256	0.5	4–128	8	32
AMP	256 – > 256	>256	>256	2	32 – > 256	128	>256
CIP	1 – > 32	8	>32	0.0625	0.0625–2	0.125	1
CRO	2 – > 32	16	32	0.0625	0.0625–2	0.25	2

*AZM, azithromycin; AMP, ampicillin; CIP, ciprofloxacin; CRO, ceftriaxone.*

**TABLE 4:** MIC of 4 antimicrobial agents for *S. sonnei*, recipient strains and transconjugants.

Antimicrobial agent	*S. sonnei* (*n* = 10)			*E. Coli* J53 AzR	Transconjugants (*n* = 10)		
			
	MIC range	MIC_50_	MIC_90_		MIC range	MIC_50_	MIC_90_
	(μg/mL)	(μg/mL)	(μg/mL)		(μg/mL)	(μg/mL)	(μg/mL)
AZM	32 – > 256	32	128	0.5	4–256	8	64
AMP	128 – > 256	>256	>256	2	32 – > 256	64	256
CIP	0.25 – > 32	0.5	32	0.0625	0.0625– 2	0.125	0.5
CRO	0.0625 – > 32	16	>32	0.0625	0.0625–2	0.25	1

*AZM, azithromycin; AMP, ampicillin; CIP, ciprofloxacin; CRO, ceftriaxone.*

### PFGE Analysis

**FIGURE 2:**
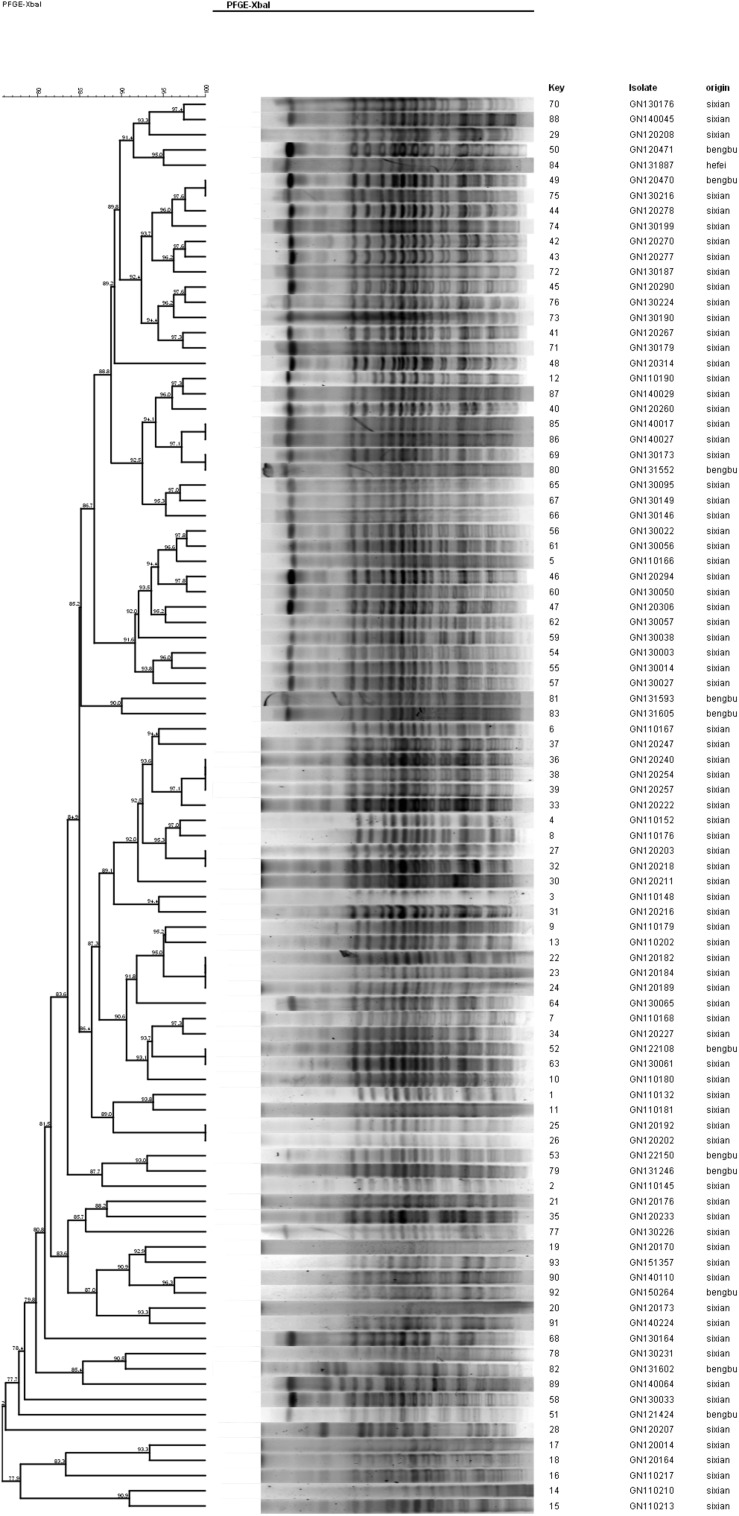
Dendogram of pulsed-field gel elecrophoresis for *mphA*-positive *S. flexneri* isolates.

**FIGURE 3:**
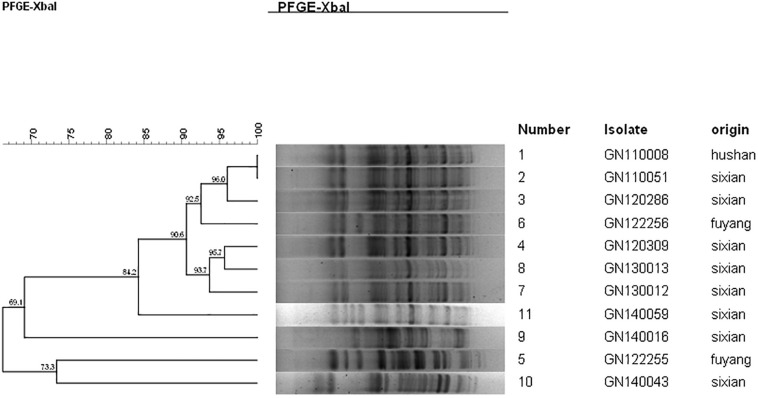
Dendogram of pulsed-field gel elecrophoresis for *mphA*-positive *S. sonnei* isolates.

The PFGE analysis was performed to determine whether clonal dissemination of *mphA*-positive *Shigella* isolates was responsible for the DSA in 104 isolates (93 *S. flexneri* and 11 *S. sonnei*). Figures 2, 3 show the dendrogram with PFGE images of *mphA*-positive *S. flexneri* and *mphA*-positive *S. sonnei* isolates, respectively. Thirteen clusters were detected for *mphA*-positive *S. flexneri* isolates and five clusters were detected for *mphA*-positive *S. sonnei* isolates. The predominant clones for *mphA*-positive *S. flexneri* isolates were P1 (44.1%, 41/93); P2 (30.1%, 28/93); and P5 (6.5%, 6/93). Clones P3, P4, and P7 of *mphA*-positive *S. flexneri* comprised three isolates (3.2%) each. Clones P11 and P13 of *mphA*-positive *S. flexneri* comprised two isolates (2.2%) each. Clones P6, P8, P9, and P12 of *mphA*-positive *S. flexneri* comprised one isolate (1.1%) each. The predominant clone for *mphA*-positive *S. sonnei* was P1 (63.6%; 7/11). Clones P2, P3, P4, and P5 of *mphA*-positive *S. sonnei* comprised one isolate (9.1%) each.

**FIGURE 4:**
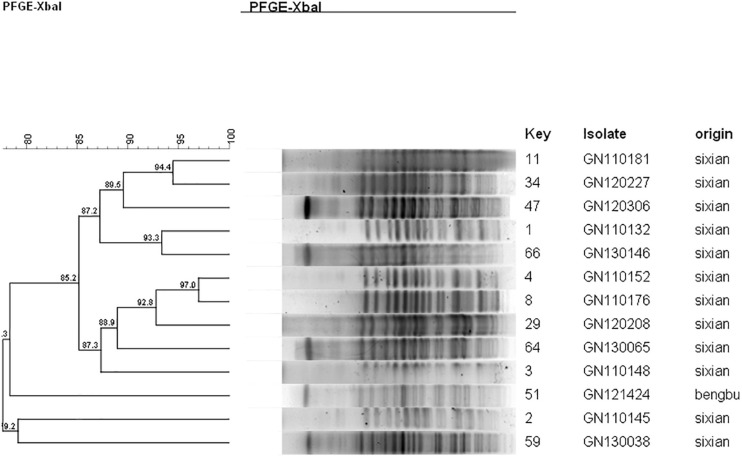
Dendogram of pulsed-field gel elecrophoresis for *S. flexneri* with high-level decreased susceptibility to azithromycin.

Ten isolates showed homology among 13 *mphA*-positive *S. flexneri* isolates with high-level DSA (Figure 4). The homology findings for the 10 *S. flexneri* isolates are presented in Table 5. All 10 homologous *S. flexneri* isolates were from Sixian county and were multidrug-resistant (MDR) strains. Of the 10 homologous *S. flexneri* isolates, eight were from children (≤5 years old), and two were from the elderly (>60 years old).

**TABLE 5:** Homologous strain information for *mphA*-positive *S. flexneri* with high-level DSA.

Isolate	Age	Sex	Year	MIC (μg/mL)				MDR	Origin	Strain
				
				AZM	CIP	CRO	AMP			
GN110132	4	m	2011	>256	16	32	>256	Yes	Sixian	1
GN110148	2	m	2011	>256	8	16	>256	Yes	Sixian	3
GN110152	82	f	2011	>256	8	32	>256	Yes	Sixian	4
GN110176	1	m	2011	>256	8	32	>256	Yes	Sixian	8
GN110181	5	m	2011	>256	8	16	>256	Yes	Sixian	11
GN120208	2	f	2012	>256	16	32	>256	Yes	Sixian	29
GN120227	4	m	2012	>256	8	16	>256	Yes	Sixian	34
GN120306	3	m	2012	>256	16	16	>256	Yes	Sixian	47
GN130065	1	f	2013	>256	16	16	>256	Yes	Sixian	64
GN130146	63	m	2013	>256	16	16	>256	Yes	Sixian	66

*DSA, decreased susceptibility to azithromycin; MIC, minimal inhibitory concentration; AZM, azithromycin; AMP, ampicillin; CIP, ciprofloxacin; CRO, ceftriaxone; MDR, multidrug-resistant.*

## Discussion

In the current study, 449 *S. flexneri* isolates showed a high resistance pattern to CIP (49.7%) and CRO (45.7%), as well as DSA (33.6%); compared with 68 *S. sonnei* isolates showing resistance to CIP (19.1%) and CRO (33.8%), as well as DSA (39.7%). These results indicate that antimicrobial resistance in *Shigella* isolates is a serious challenge in Anhui, China. It is notable that DSA has increased globally and antimicrobial resistance among *Shigella* isolates can vary from region to region. For example, in India, the DSA rate was 48% in *Shigella* isolates from 2006 to 2011 (Bhattacharya et al., 2014). In Palestine, the DSA rate was 42% in *Shigella* isolates from 2004 to 2014 (Salah et al., 2019). However, in Australia, the DSA rate was 13.1% in *Shigella* isolates from 2013 to 2014, and the DSA has been reported more frequently in men who have sex with men (MSM) (Brown et al., 2017). In the United States, the DSA rate of *Shigella* isolates was 4.2% in 2012 (Sjölund et al., 2013).

In the current study, no significant differences were noted in gender and age between *S. flexneri* and *S. sonnei* isolates with DSA (Table 1). However, some studies have shown that males are more likely to be infected with a DSA strain than females, especially the MSM (Baker et al., 2015; Liao et al., 2016; Brown et al., 2017). The most likely explanation for this findings is the fact that *Shigella* isolates in the current study were isolated mainly from children < 5 years old. There are no clinical breakpoints for AZM in *Shigella* species in the CLSI guidelines. According to the CLSI (2019), the ECVs of MICs ≥ 16 μg/mL (for *S. flexneri*) and ≥ 32 μg/mL (for *S. sonnei*) are considered indicative of DSA, which is supported by the current results. In our data, *S. flexneri* (MIC_50_ 4 μg/mL) had a lower MIC distribution than *S. sonnei* (MIC_50_ 16 μg/mL).

Acquired macrolide resistance may arise from various mechanisms. The plasmid-mediated resistance determinants play an important role in the prevalence of DSA isolates because they are readily transferable. In the current study, 93 isolates (57.8%) carried the *mphA* gene among 161 DSA *S. flexneri* isolates, and 11 isolates (40.7%) carried the *mphA* gene among 27 DSA *S. sonnei* isolates. No other plasmid-mediated DSA genes were detected. Interestingly, all *S. flexneri* isolates with an MIC > 256 μg/mL carried the *mphA* gene. This indicates that the *mphA* gene is closely related to a high level of DSA (MIC > 256 μg/mL).

Furthermore, the *mphA* gene is normally associated with mobile elements (Gaudreau et al., 2014; Darton et al., 2018; Yousfi et al., 2019). Therefore, it was interesting to determine whether *mphA*-positive *Shigella* isolates would be able to transfer their macrolide resistance genes to recipients, and if the resulting transconjugants would be macrolide-resistant. In the current study, 89 transconjugants for *mphA*-positive *S. flexneri* and 10 transconjugants for *mphA*-positive *S. sonnei* were obtained by conjugation. This indicates that the *mphA* gene could be transferred between bacteria by horizontal exchange.

Furthermore, the transconjugants of *mphA*-positive *S. flexneri* isolates showed a 32-fold increase in the MIC_50_ and 128-fold increase in the MIC_90_ of AZM, compared with the recipient (Table 3). The transconjugants of *mphA*-positive *S. sonnei* isolates showed a 16-fold increase in the MIC_50_ and 128-fold increase in the MIC_90_ of AZM, compared with the recipient (Table 4). The above results are consistent with those of *E. coli* transconjugants with elevated MICs of AZM, and the confirmed location of the *mphA* gene on a plasmid (Sjölund et al., 2013).

Despite the high diversity, 80.7% of the *mphA*-positive DSA *S. flexneri* isolates were represented by three dominant PFGE clusters (P1, P2, and P5), and 63.6% of the *mphA*-positive DSA *S. sonnei* isolates were represented by one dominant PFGE cluster (P1). A genetic similarity value of 85% could indicate clonal spread of *mphA*-positive DSA-*Shigella* isolates in the community. Furthermore, genetic similarity was noted between the *mphA*-positive *Shigella* isolates with high-level DSA that was transmitted during the years 2011–2013.

Notably, all 10 homologous *mphA*-positive *S. flexneri* isolates with high-level DSA were MDR strains and were from children and the elderly. These findings might be attributed to a poor immune status in children and the elderly. These results also indicate that the *mphA*-positive *S. flexneri* isolates with high-level DSA were associated with clonal spread in children and the elderly.

Recently, researchers found that shigellosis can be transmitted among MSM patients, in whom oroanal sex and concurrent infection with HIV can significantly increase the infection rate (Baker et al., 2015; Liao et al., 2016; Yousfi et al., 2019). Baker et al. (2015) described the intercontinental dissemination of DSA shigellosis in MSM through sexual transmission. However, this concept was not analyzed in the current study, because data on MSM patients was not collected from an early stage. In future research, we will pay attention to the spread of DSA shigellosis in MSM in Anhui, China. *Shigella* isolates with DSA remains a topic of great concern. A provincial surveillance program should be established to determine the prevalence of *Shigella* with DSA. Furthermore, studies should be extended to elucidate the transmission mechanisms associated with DSA, in order to limit and prevent its emergence.

## Conclusion

The current study demonstrates that the DSA for *Shigella* isolates is serious. We also confirmed that the plasmid-mediated *mphA* gene is the most common macrolide resistance gene in *Shigella* isolates collected in Anhui, China. The plasmid-mediated *mphA* gene can be transferred horizontally, to yield either the same or different strains with DSA. Our results also suggest the presence of a high diversity of *mphA*-positive DSA-*Shigella* isolates. It was noteworthy that the *mphA*-positive *S. flexneri* isolates with high-level DSA was associated with clonal spread in children and the elderly. This finding warrants further attention.

## Data Availability Statement

All datasets generated for this study are included in the article/Supplementary Material.

## Ethics Statement

The studies involving human participants were reviewed and approved by the local Ethics Committees of the First Affiliated Hospital of Anhui Medical University (Hefei, China). Written informed consent to participate in this study was provided by the participants’ legal guardian/next of kin.

## Author Contributions

JL designed the experiments. YL, HL, and NL performed the experiments. XX, YZ, YY, and YG analyzed the results. All authors involved in the design of the experiments and reviewed the manuscript.

## Conflict of Interest

The authors declare that the research was conducted in the absence of any commercial or financial relationships that could be construed as a potential conflict of interest.

## Abbreviations

AMP: ampicillin
AZM: azithromycin
CIP: ciprofloxacin
CRO: ceftriaxone
DSA: decreased susceptibility to azithromycin
MDR: multidrug-resistant
MIC: minimal inhibitory concentration.
