# Supplementary material for: Prevalence of Plasmid-Mediated Determinants With Decreased Susceptibility to Azithromycin Among Shigella Isolates in Anhui, China
Source: Front Microbiol. 2020 Jun 30;11:1181. doi: 10.3389/fmicb.2020.01181 (PMC7338677; doi:10.3389/fmicb.2020.01181)
Supplement: Supplementary file 1 [file Table_1.DOCX]

**Table S1. Oligonucleotide primer sequences used for PCR amplification**

| Target  gene | Primer | Sequence (5'-3') | Product size(bp) | Annealing  temperature(℃) |
| --- | --- | --- | --- | --- |
| mph(A) | mphAF | GTG AGG AGG AGC TTC GCG AG | 403 | 60 |
|  | mphAR | TGC CGC AGG ACT CGG AGG TC |  |  |
| mph(B) | mphBF | GAT ATT AAA CAA GTA ATC AGA ATA G | 494 | 58 |
|  | mphBR | GCT CTT ACT GCA TCC ATA CG |  |  |
| erm(A) | ermAF | TCT AAA AAG CAT GTA AAA GAA A | 533 | 52 |
|  | ermAR | CGA TAC TTT TTG TAG TCC TTC |  |  |
| erm(B) | ermBF | GAA AAA GTA CTC AACCAAATA | 639 | 45 |
|  | ermBR | AAT TTA AGT ACC GTT ACT |  |  |
| erm(C) | ermCF | TCA AAA CAT AAT ATA GAT AAA | 642 | 45 |
|  | ermCR | GCT AAT ATT GTT TAA ATC GTC AAT |  |  |
| erm(F) | ermFF | CGA CAC AGC TTT GGT TGA AC | 309 | 56 |
|  | ermFR | GGA CCT ACC TCA TAG ACA AG |  |  |
| erm(T) | ermTF | CAT ATA AAT GAA ATT TTG AG | 369 | 51 |
|  | ermTR | ACG ATT TGT ATT TAG CAA CC |  |  |
| erm(X) | ermXF | GAG ATC GGR CCA GGA AGC | 488 | 58 |
|  | ermXR | GTG TGC ACC ATC GCC TGA |  |  |
| ere(A) | ereAF | GCC GGT GCT CAT GAA CTT GAG | 420 | 60 |
|  | ereAR | CGA CTC TAT TCG ATC AGA GGC |  |  |
| ere(B) | ereBF | TTG GAG ATA CCC AGA TTG TAG | 537 | 55 |
|  | ereBR | GAG CCA TAG CTT CAA CGC |  |  |
| mef(A) | mefAF | AGT ATC ATT AAT CAC TAG TGC | 345 | 54 |
|  | mefAR | TTC TTC TGG TAC TAA AAG TGG |  |  |
| msr(A) | msrAF | GCA CTT ATT GGG GGT AAT GG | 384 | 58 |
|  | msrAR | GTC TAT AAG TGC TCT ATC GTG |  |  |
